# Jumping without slipping: leafhoppers (Hemiptera: Cicadellidae) possess special tarsal structures for jumping from smooth surfaces

**DOI:** 10.1098/rsif.2017.0022

**Published:** 2017-05-03

**Authors:** Christofer J. Clemente, Hanns Hagen Goetzke, James M. R. Bullock, Gregory P. Sutton, Malcolm Burrows, Walter Federle

**Affiliations:** 1 Department of Zoology, University of Cambridge, Cambridge CB2 3EJ, UK; 2 School of Biological Sciences, University of Bristol, Bristol BS8 1UG, UK

**Keywords:** jumping insects, controllable attachment, anisotropic friction, adhesion, biomechanics, kinematics

## Abstract

Many hemipteran bugs can jump explosively from plant substrates, which can be very smooth. We therefore analysed the jumping performance of froghoppers (*Philaenus spumarius,* Aphrophoridae) and leafhoppers (*Aphrodes bicinctus/makarovi,* Cicadellidae) taking off from smooth (glass) and rough (sandpaper, 30 µm asperity size) surfaces. On glass, the propulsive hind legs of *Philaenus* froghoppers slipped, resulting in uncontrolled jumps with a fast forward spin, a steeper angle and only a quarter of the velocity compared with jumps from rough surfaces. By contrast, *Aphrodes* leafhoppers took off without their propulsive hind legs slipping, and reached low take-off angles and high velocities on both substrates. This difference in jumping ability from smooth surfaces can be explained not only by the lower acceleration of the long-legged leafhoppers, but also by the presence of 2–9 soft pad-like structures (platellae) on their hind tarsi, which are absent in froghoppers. High-speed videos of jumping showed that platellae contact the surface briefly (approx. 3 ms) during the acceleration phase. Friction force measurements on individual hind tarsi on glass revealed that at low sliding speeds, both pushing and pulling forces were small, and insufficient to explain the recorded jumps. Only when the tarsi were pushed with higher velocities did the contact area of the platellae increase markedly, and high friction forces were produced, consistent with the observed jumps. Our findings show that leafhoppers have special adhesive footpads for jumping from smooth surfaces, which achieve firm grip and rapid control of attachment/detachment by combining anisotropic friction with velocity dependence.

## 1. Introduction

Many insects jump to escape from predators, to move in complex terrain or to launch into flight. Some of the most proficient jumping insects are found among plant-sucking bugs in the hemipteran sub-order Auchenorrhyncha, including froghoppers [1–3] and leafhoppers [4–7]. In contrast to insects with very long hind legs that power their jumps mainly by direct muscle action (e.g. bush crickets, [8]), jumping Auchenorrhyncha have shorter hind legs and employ catapult mechanisms to propel themselves off the ground [9–14]. In these jumps, the acceleration typically lasts only a few milliseconds. How are insects able to transmit forces to the ground during this short time?

Many jumping hemipteran and orthopteran insects are generalist herbivores that live and feed on multiple species of plants [15,16], which can have microscopically smooth surfaces [17,18]. To jump forward, they have to produce high forces parallel to the ground. On a rough substrate, spines or claws on the hind legs may be able to grip, but they may not be able to engage with smooth surfaces [19,20]. Insects also cannot rely on classic friction alone, because their take-off angle *α* = tan^−1^(*F*_normal_/*F*_shear_) = tan^−1^(1/*μ*) is limited by the friction coefficient μ (Amontons' law of friction: *F*_shear_ = **μ* F*_normal_, where *F*_shear_ is the force parallel to the surface and *F*_normal_ the load normal to the surface). Friction coefficients *μ* for rigid, dry surfaces are typically less than 1. Assuming **μ** = 0.35 (for beetle claws on glass [19]), insects could only jump upward with steep take-off angles *α* > 70°. To jump forward, insects require significantly higher friction coefficients (**μ**), which could be achieved by adhesive structures that strengthen the surface contact when they accelerate before take-off. However, the use of adhesive structures for jumping comes with several biomechanical challenges. Firstly, if the feet of the propulsive legs adhered too well to the surface, they would slow down the jump. As insect jumps are very brief, an extremely rapid control of surface adhesion would be required.

Secondly, most adhesive devices used by climbing insects are directional, i.e. they stick when legs are pulled towards the body but detach when pushed, thereby allowing easy detachment during locomotion (e.g. flies: [21]; bush crickets: [22]; ants: [23,24]; beetles: [25]; stick insects: [25,26]). However, jumping requires the hind legs to push against the typical gripping direction of the distal adhesive pads. Some insects possess tarsal pads which are specialized for pushing and/or generating high friction forces [27–30] but no such structures are known for hemipterans or other jumping insects.

Insect jumping performance has been studied mostly by allowing insects to jump from rough substrates such as twigs, high-density foam or sandpaper [3,5,31,32]. Here we study whether and how *Philaenus spumarius* froghoppers (Aphrophoridae) and *Aphrodes bicinctus/makarovi* leafhoppers (Cicadellidae) can jump from smooth surfaces. *Philaenus* frog-hoppers have relatively short hind legs (66% of the body length), are able to accelerate in less than 1 ms to take-off velocities of up to 4.7 m s^−1^, when jumping from high-density foam [3]. In comparison, *Aphrodes* leafhoppers have longer hind legs (84% of the body length), take longer (4.4 ms) to accelerate and achieve take-off velocities of up to 2.9 m s^−1^ on high-density foam [5].

The mechanisms and potential adaptations of insects for jumping from smooth surfaces have not been studied. Here, we address the following questions: (i) are *Aphrodes* leafhoppers and *Philaenus* froghoppers able to jump from smooth surfaces? (ii) Does jumping performance differ between smooth and rough surfaces? (iii) What structures come into surface contact during take-off and how are they adapted? (iv) How is attachment and detachment controlled in these structures?

## 2. Material and methods

### 2.1. Study animals

We studied two hemipterans of similar size, the leafhopper *Aphrodes* of the *bicinctus* Schrank 1776/*makarovi* Zachvatkin 1948 group (Cicadellidae) and the froghopper *Philaenus spumarius* Linnaeus 1758 (Aphrophoridae). We collected 31 adult *A. bicinctus/makarovi* (body mass 18.4 ± 0.6 mg, mean ± standard error of the mean) and 43 adult *P. spumarius* (13.3 ± 0.4 mg) on meadows around Cambridge (UK) from several species of plants. Both insects have been recorded to live on diverse host plants [33,34]. Observations on live insects were made within 1 day of collection.

### 2.2. Morphology

The tarsal morphology of leafhoppers and froghoppers was examined in a Leica MZ 16 stereo microscope (Leica Microsystems GmbH, Wetzlar, Germany). For scanning electron microscopy (SEM), legs were fixed in 4% glutaraldehyde in 0.1 M PIPES buffer at pH 7.4 for 48 h at 4°C. They were then washed with de-ionized water and dehydrated in increasing concentrations of ethanol (up to 96% ethanol). The legs were air-dried and mounted on stubs, sputter coated with a 20 nm layer of gold, and examined in a FEI XL 30-FEG SEM (FEI, Hillsboro, USA) at 10 kV.

### 2.3. Jumping performance from smooth and rough surfaces

To compare jumping performance on smooth and rough surfaces, insects were placed on a smooth glass coverslip (18 × 18 × 0.17 mm) or on sandpaper (glued onto an 18 × 18 mm plate) with 30 μm nominal asperity size. If the insects did not jump spontaneously, they were carefully prompted using a fine natural hair paintbrush. Jumps were recorded with two high-speed video cameras positioned to achieve dorsal and lateral views, either at 1000 frames per second (fps) using two Redlake PCI 1000 B/W (Redlake Imaging, San Diego, CA, USA) or at 4700 fps using two Phantom v. 7.1 (Vision Research, Wayne, NJ, USA).

Each recorded jump was analysed using a custom-written Matlab script (Mathworks, Natick, MA, USA) by digitising the insects' body position in both camera views; take-off angle and velocity were calculated trigonometrically from the flight trajectory over the first 2 ms in which the insect was airborne.

### 2.4. Contact area recordings during jumps from glass

To record the contact areas of hind tarsi during the acceleration phase of a jump (defined as the time between the first visible hind leg movement and take-off), *Aphrodes* leafhoppers and *Philaenus* froghoppers were placed on glass coverslips on an inverted Leica DM IRE2 microscope. Contact areas were visualized using a 5× lens and bright field epi-illumination from a 100 W mercury arc lamp. This illumination produces high-contrast images of the pad contact areas [35]. Contact areas of hind tarsi before take-off were recorded in *Aphrodes* using a FASTCAM 1024 PCI high-speed camera (Photron, San Diego, CA, USA) at 5000 fps. Contact areas and a simultaneous close-up side view of the insect were recorded in *Aphrodes* and *Philaenus* using two Phantom v. 7.1 high-speed cameras (Vision Research, Wayne, NJ, USA) at 4700 fps. In total, we obtained high-speed close-up recordings of the hind tarsi for 12 jumps of five *Aphrodes* and nine jumps of eight *Philaenus*.

In most of the recordings, contact areas of only one hind leg was visible. For each jump, the contact area was measured using a threshold algorithm in Matlab. In jumps of *Aphrodes*, velocities along the sliding trajectory of platellae in contact with the glass coverslip were measured by manually digitising the proximal end of the platella contact area three times (to reduce the measurement error) using ImageJ. We used the average of the three measured values for each frame. As we digitised the proximal end of the platella contact zone, it is possible that we slightly underestimated the sliding distance (the contact area expands mainly by growing on its proximal side). The first frame without visible surface contact of the hind tarsi was defined as ‘take-off’ and the time as 0 ms (note that when hind legs slipped, the front legs usually remained in contact for a few frames after take-off).

### 2.5. Single leg friction force measurements

Friction forces of the hind tarsi of *Aphrodes* and *Philaenus* on glass were measured using a two-dimensional force transducer mounted on a three-dimensional motor positioning stage. A custom LabVIEW (National Instruments, Austin, TX, USA) program collected data and controlled movements of the force transducer and video trigger signals (for details of the force measurement set-up, see [36]). The hind leg of a live insect was mounted in Blu Tack and the ventral side of the first two tarsal segments was brought into contact with a glass coverslip at the end of the force transducer. To prevent the claws and arolium from making contact, the pretarsus was bent away and fixed with Blu Tack. Contact area was recorded under reflected light using a Redlake PCI 1000 B/W at 10 fps. To measure the contact area, a greyscale threshold was determined in a region of interest around the contact area using Matlab's inbuilt *graythresh* function. Using this threshold value and filtering the detected area with Matlab's two-dimensional median filter *medfilt2*, the contact area was measured.

In previously recorded jumps of *Philaenus*, mean forces of 34 mN were found in the direction of the jump with a mean take-off angle of 46.8° [3]. Such a jump would produce a normal force of 24.8 mN or 12.4 mN per hind leg. For *Aphrodes*, mean forces of 11 mN were found with a mean take-off angle of 37.1° [5], giving a mean normal force of 6.6 mN or 3.3 mN per hind leg. One of the insect's hind legs was brought into contact with the glass coverslip with a feedback-controlled normal force of 5 mN. To investigate the effect of normal forces, we also conducted comparable measurements with a normal force of 3 and 1 mN for the same froghoppers and leafhoppers. While the normal force was kept constant, the force transducer was moved horizontally for 2 s at sliding velocities ranging from 0.1 to 5 mm s^−1^ in random order and at different positions on the glass coverslip to avoid accumulation of adhesive secretion [25,36]. These experimentally applied velocities were well below those measured for leafhoppers during natural take-off; velocities higher than 5 mm s^−1^ could not be tested with our set-up. For each velocity, slides were performed in both the pushing and pulling direction (corresponding to leg movements away from or towards the body, respectively) in random order. After each slide, the foot was left in contact for 2 s before a 0.5 mm s^−1^ pull-off in the normal direction. The noise level of the measurement set-up for both adhesion and friction was less than 0.2 mN.

Statistical analysis of the data was performed in R v. 3.0.2 [37]. Data are given as mean ± standard error of the mean (s.e.m.) unless specified otherwise. Linear mixed effects models were performed using the R package *nlme* [38] and Page's L trend test was performed using the package *crank* [39].

## 3. Results

### 3.1. Morphology

**Figure 1.**
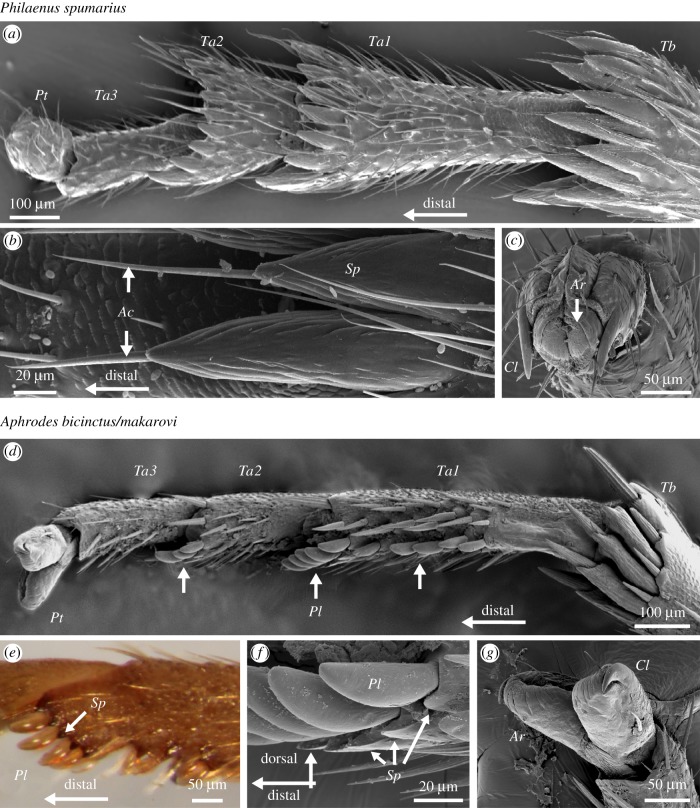
Hind leg tarsi of *Philaenus spumarius* (*a*–*c*) and *Aphrodes bicinctus/makarovi* (*d*–*g*), scanning electron micrographs (except *e*). (*a*) *Philaenus*, ventral view of distal tibia and tarsus. (*b*) Ventral view of spines on the distal end of the tibia. A single hair (acutella) protrudes from below each spine. (*c*) Arolium on the pretarsus. (*d*) *Aphrodes*, ventro-lateral view of distal tibia and tarsus, with platellae (white arrows) on tarsomere 1 and tarsomere 2. (*e*) Platellae are brighter and less sclerotized than the surrounding cuticle of tarsomere 1. (*f*) Row of platellae on distal end of tarsomere 1. (*g*) Bi-lobed arolium on the pretarsus. *Ac*: acutella; *Ar*: arolium, *Cl*: claw, *Pl*: platella, *Pt*: pretarsus, *Sp*: spine; *Ta1*: tarsomere 1, *Ta2*: tarsomere 2, *Ta3*: tarsomere 3, *Tb*: tibia.

The tarsus of leafhoppers and froghoppers consists of three segments and a distal pretarsus containing claws and a bi-lobed adhesive pad (arolium) (figure 1). The hind but not the two other pairs of legs of both *Philaenus* froghoppers and *Aphrodes* leafhoppers possess rows of conical, sclerotized spines ventrally at the distal end of their hind tibia. In *Philaenus*, these spines do not articulate with the tibia, but in *Aphrodes* they are hinged. *Philaenus* has similar rows of sclerotized spines also on the first and second tarsomeres but *Aphrodes* has only individual small spines.

In *Philaenus*, a single flexible hair (‘acutella’ [40]), 60 to 165 μm long and 4 to 10 μm wide at its base (*n* = 135 hairs of *N* = 9 animals), protrudes from the dorsal side of each spine (figure 1*b*). By contrast, the tarsus of *Aphrodes* bears many conspicuous, rounded cuticular outgrowths (‘platellae’, [40–42]), which also emerge from the dorsal surface of the base of the spines; five to six at the distal end of the first tarsomere, up to eight in a row along the first tarsomere, and two to three at the distal end on the second tarsomere (*N* = 8 animals) (figure 1*d–f*). They are 65–100 μm long (measured from the base, 13 platellae from *N* = 5 animals) and less sclerotized than the surrounding cuticle with a pale, yellowish colour (figure 1*e*). In lateral view, they appear approximately straight on the dorsal side, but convex on the ventral side (figure 1*f*).

### 3.2. Jump performance on smooth and rough surfaces

**Figure 2.**
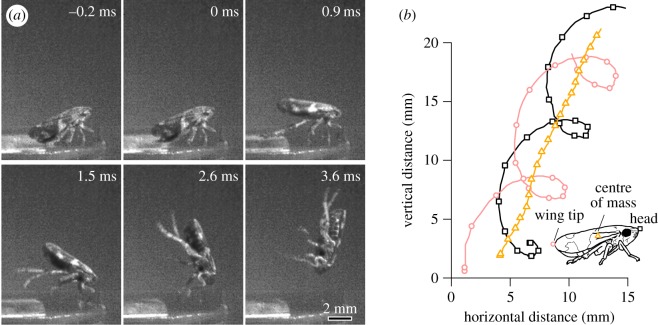
(*a*) Selected video frames of an attempted jump of *Philaenus spumarius* from glass recorded at 4700 fps. The hind legs detached at 0 ms and the front legs at 0.9 ms. (*b*) Trajectories of the head, wing tip and centre of mass. Points show every fifth tracked position (corresponding to 1.1 ms).

**Figure 3.**
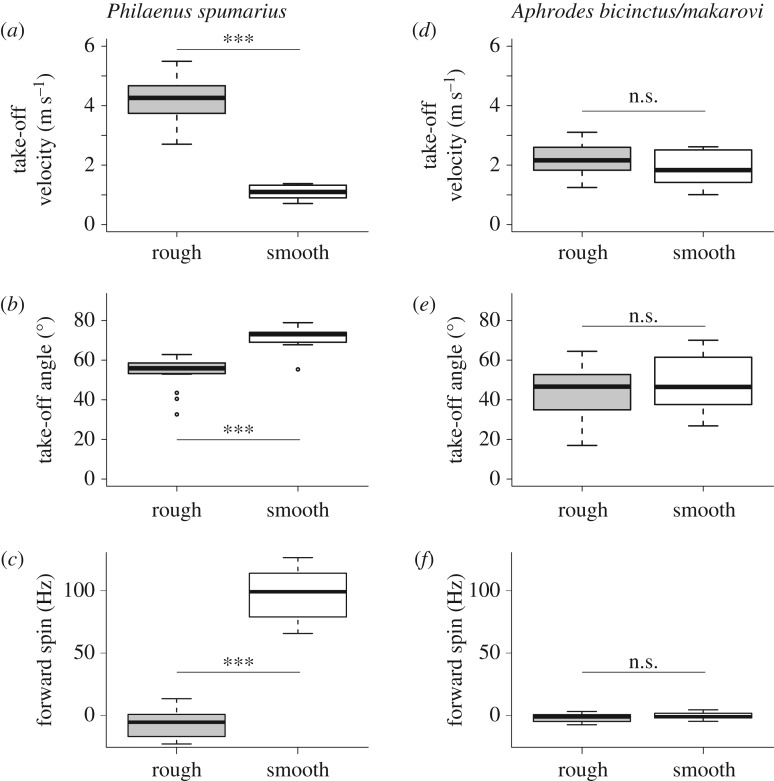
Jump performance from rough and smooth surfaces in *Philaenus spumarius* froghoppers (*a*–*c*) and *Aphrodes* leafhoppers (*d*–*f*). Take-off velocity (*a*,*d*), take-off angle (*b*,*e*), and forward spin (*c*,*f*).***: *p* < 0 : 001, n.s.: no significant difference.

When *Philaenus* jumped from smooth glass their hind legs always slipped (figure 2, electronic supplementary material, video S1) whereas slipping never occurred on the rough sandpaper (electronic supplementary material, video S2). As a result of the slipping, the take-off velocity of *Philaenus* on glass was only one quarter of that on sandpaper (sandpaper: 4.2 ± 0.2 m s^−1^, *N* = 16 insects; glass: 1.1 ± 0.1 m s^−1^, *N* = 10 insects, Welch's *t*-test: *t*_19.5_ = 16.2, *p* < 0.001; figure 3*a*). The slipping of the propulsive hind legs on glass resulted in significantly steeper jumps (take-off angle on glass 71.3 ± 2.0°, *N* = 10 insects; sandpaper: 53.8 ± 2.1°, *N* = 16 insects; Wilcoxon rank sum test: *W* = 9, *p* < 0.001; figure 3*b*) and a rapid forward spin (clockwise in figure 2; 97.2 ± 6.6 Hz, *N* = 10 insects), whereas *Philaenus* jumping from sandpaper showed a weak backspin (−7.34 ± 2.54 Hz, *N* = 16 insects; Welch's *t*-test: *t*_11.7_ = 14.8, *p* < 0.001; figures 2*b* and 3*c*). Both the steeper take-off angle and the forward spin result from the near-complete loss of forward thrust when the hind legs slip. In normal jumps without slipping, the hind legs push both backward (parallel to the surface) and downward (perpendicular to the surface), but they can only push downward when they slip, thereby accelerating the insect's rear end upward. This produces a forward rotation of the insect's body around the front feet (while these are still in contact), accelerating the body centre of mass upward (figure 2). Once the insect is completely airborne, the forward spin continues around the body centre of mass. Even with the rotational energy included, the total kinetic energy of the jump was nine-times smaller on glass than on sandpaper (glass: 13.3 ± 1.6 μJ, *N* = 10 insects, sandpaper: 121.8 ± 10.1 μJ, *N* = 16 insects; Welch's *t*-test: *t*_15.7_ = 10.6, *p* < 0.001), indicating that most of the energy is dissipated by the slipping and kicking hind legs [43].

In contrast with *Philaenus*, we never observed *Aphrodes* slip on any of the substrates (electronic supplementary material, videos S3 and S4). Consistently, their take-off velocity did not depend significantly on the substrate (sandpaper: 2.2 ± 0.1 m s^−1^, *N* = 18 insects; glass: 1.9 ± 0.2 m s^−1^, *N* = 10 insects; Welch's *t*-test: *t*_26_ = 1.7, *p* = 0.111; figure 3*d*) and they jumped with similar take-off angles (sandpaper: 44.7 ± 2.7°; glass: 47.7 ± 4.4°; Welch's *t*-test: *t*_26_ = 0.6, *p* = 0.552; figure 3*e*). The jumps of *Aphrodes* showed only minimal backspin on both substrates (sandpaper: 2.13 ± 0.90 Hz, *N* = 13 leafhoppers; glass: 0.69 ± 0.97 Hz, *N* = 9 leafhoppers; Welch's *t*-test: *t*_18.5_ = 1.1, *p* = 0.291; figure 3*f*). The total kinetic energy was similar for jumps on both surfaces (sandpaper: 49.3 ± 6.5 μJ, *N* = 13 insects; glass: 36.8 ± 6.5 μJ, *N* = 9 insects, Welch's *t*-test: *t*_19.2_ = 1.4, *p* = 0.188).

### 3.3. Contact area recordings during jumps from glass

**Figure 4.**
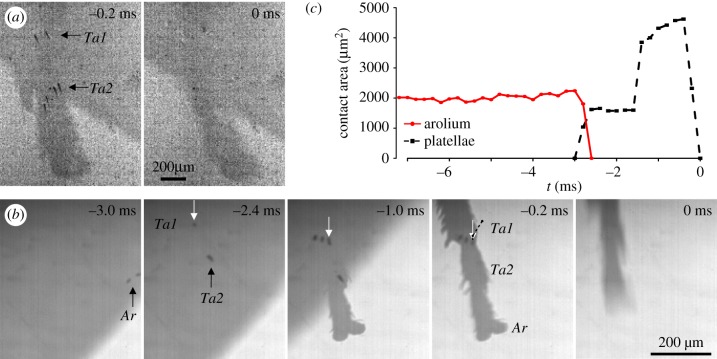
(*a*) Contact area images during a jump of *Philaenus spumarius* recorded in reflected-light illumination (see also electronic supplementary material, video S5)*.* Black arrows mark the hairs (acutellae) on the hind leg tarsus, which detached with the first visible leg movement. (*b*) Sequential images of the hind leg tarsus during a jump of *Aphrodes bicinctus/makarovi* (see also electronic supplementary material, video S6)*.* Because of the reflected-light illumination, areas in contact with glass appear dark. The white arrows in images 2–4 mark the same platella; note that its contact area increased strongly and that additional platellae came into contact from −2.4 to −1.0 ms. The black dotted line in frame ‘−0.2 ms’ marks the sliding trajectory of the platella marked by the white arrows. (*c*) Changes in the contact area of the arolium and all the platellae before take-off. Take-off (0 ms) is defined as the first frame without visible contact area; the first visible hind leg movement occurred at −2.8 ms. *Ar*: arolium, *Ta1*: tarsomere 1, *Ta2*: tarsomere 2.

Before take-off from glass, fine hairs on the hind tarsi of *Philaenus* were observed in contact with the glass surface (figure 4*a*; electronic supplementary material, video S5). Within the first two frames of visible hind leg movements, however, these hairs were already detached and remained out of contact while the leg was slipping. In eight out of nine jumps the hind leg arolium was in contact before the jump, but also detached at the start of the acceleration phase.

Contact area recordings for the hind legs of jumping *Aphrodes* (12 jumps by five animals) showed that two to nine platellae on the first and second tarsomere came into surface contact at the start of the acceleration phase (when the first hind leg movements became visible) and remained in contact until take-off (figure 4*b,c*; electronic supplementary material, video S6); the mean contact area was 2484 ± 258 *μ*m^2^ (averaged across individuals and acceleration time). The platellae were in surface contact for 3.1 ± 0.1 ms. Contact area increased rapidly at the beginning of the contact phase, both by expansion of the contact area of individual platellae (see the platella marked by the arrow in figure 4*b*,*c*) and by additional platellae coming into contact (figure 4*b*,*c*; electronic supplementary material, figure S1*a*). Contact areas decreased rapidly 0.6 to 0.2 ms before take-off. In three jumps (of two animals), hind leg arolia were in surface contact before the jump, but their contact area strongly decreased or they detached at the beginning of the acceleration phase.

During the brief contact phase, the platellae slid backwards (against the direction of the jump) over a short distance (electronic supplementary material, video S6). The sliding distance ranged from 7 to 358 μm (median 41 μm, *N* = 12 jumps of five leafhoppers), corresponding to up to 5.3 times the maximal length of the contact area of one platella (maximal length of the contact area of one platella ranged from 32 to 67 μm in, *N* = 12 jumps of five leafhoppers). Platellae slid fastest at the start of the contact phase, with peak velocities ranging from 15 to 154 mm s^−1^, and then slowed down or completely stopped before take-off. In some jumps, platellae even moved slightly in the direction of the jump just before detachment (electronic supplementary material, figure S1*b*). Higher velocities of greater than 160 mm s^−1^ were sometimes observed when the platellae came into initial contact, but the contact areas were blurred, suggesting incomplete surface contact.

### 3.4. Single leg friction force measurements

Friction forces of single hind leg tarsi on glass were measured in the pulling and pushing direction at varying sliding velocities and normal forces for both *Aphrodes* and *Philaenus*.

**Figure 5.**
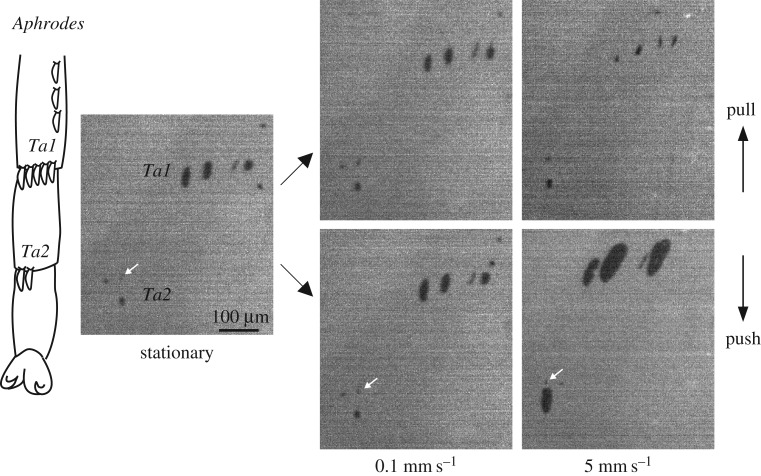
Contact area of hind leg platellae of *Aphrodes* during shear force measurements at different sliding velocities. Platellae were brought into contact with a normal force of 5 mN (‘stationary’) and then sheared in the pushing and pulling direction for 2 s, while keeping the normal force constant. Images show contact areas 0.2 s before the end of the sliding movement. *Ta1*: tarsomere 1, *Ta2*: tarsomere 2. White arrows show the tip of the tarsal spine adjacent to the platella on *Ta2*. It can be seen that the contact area of the platella expanded both laterally and longitudinally by elongating mainly on the proximal side. The scale bar applies to all images.

**Figure 6.**
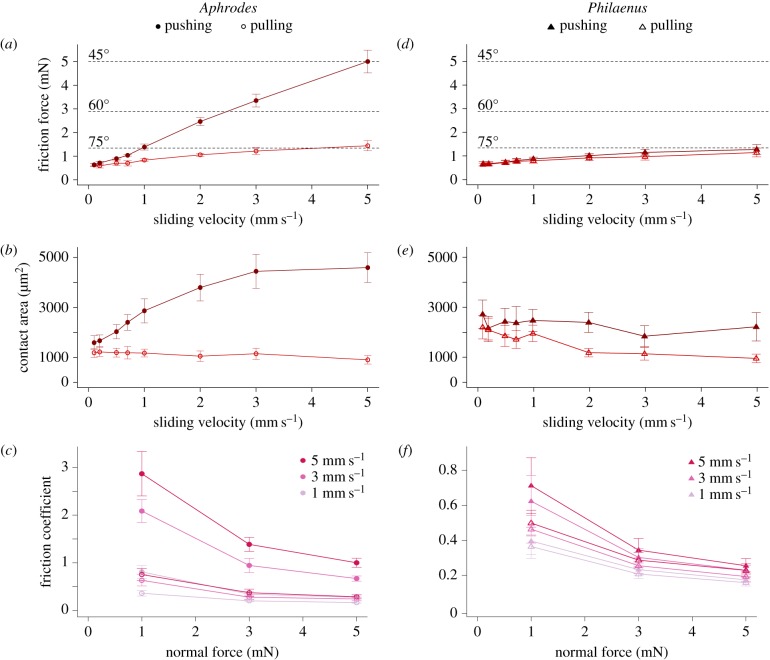
Results of force measurements on the hind leg tarsi of *Aphrodes bicinctus/makarovi* leafhoppers (*a*–*c*) and *Philaenus spumarius* froghoppers (*d*–*f*). (*a*,*d*) Peak shear (pushing and pulling) forces for 5 mN normal load and varying sliding velocities (*Aphrodes N* = 4, *Philaenus N* = 5). (*b*,*e*) Contact areas corresponding to the peak forces in (*a*) and (*d*). (*c*,*f*) Effect of normal force on friction coefficients for three different sliding velocities (*Aphrodes N* = 4; *Philaenus N* = 5). Note the different scale of the vertical axis for both insects. Dashed lines in (*a*) and (*d*) represent the minimum shear force required to jump with a take-off angle of 45°, 60° or 75°. Filled circles indicate slides in the pushing direction, open circles in the pulling direction.

When *Aphrodes* tarsi were tested at low sliding velocities (0.1 mm s^−1^, 5 mN normal force; electronic supplementary material, video S7), the total contact area of the platellae was small and only slightly larger in the pushing direction (mean area, pushing direction: 1591 ± 279 μm^2^; pulling direction: 1188 ± 184 μm^2^; paired *t*-test: *t*_3_ = 3.5, *p* = 0.039; figures 5 and 6; electronic supplementary material, table S1). The maximum contact area in the pushing direction of individual platellae was 286 ± 70 μm^2^ (*N* = 4 animals), much smaller than the areas observed during natural jumps (1114 ± 82 μm^2^, *N* = 12 jumps of five leafhoppers). The friction forces were also small in both the pushing and pulling direction (0.6 ± 0.1 mN, *N* = 4; figure 6), implying a friction coefficient of **μ** = 0.13. This would allow only steep upward jumps with take-off angles more than 82.7°, much steeper than the insects' natural jumps.

However, a very different behaviour occurred for higher sliding velocities (5 mm s^−1^, 5 mN normal force, electronic supplementary material, video S8): when the tarsi were pushed, the contact area of each platella expanded dramatically (maximum contact area of individual platellae: 1379 ± 110 μm^2^, *N* = 4 animals; almost five-times larger than at 0.1 mm s^−1^), so that their size was consistent with that observed during natural jumps. This contact area expansion was associated with a strongly increased friction force (Page's *L* test: *L*_4,8_ = 815, *p* < 0.001; figure 6*a*). The friction coefficient for 5 mm s^−1^ velocity at 5 mN normal force was **μ** = 1.00, which would allow forward jumps with take-off angles as low as 44.9°. By contrast, the contact area during pulling decreased slightly with velocity (figure 6*b*). Nevertheless, pulling friction increased with sliding velocity (because of the increase of shear stress with velocity, see below), but forces were much lower than for pushing (figure 6*a*; electronic supplementary material, table S1).

Thus, the contact area and friction force of platellae changed both with direction and velocity. Most of the increase in contact area and friction occurred for velocities changing from 0.1 mm s^−1^ to 3 mm s^−1^, and higher velocities only led to a small additional increase (figures 5 and 6*b*). Our highest experimentally applied velocity of 5 mm s^−1^ was still lower than the naturally observed sliding velocities (see above); it is therefore likely that even higher friction coefficients are reached during natural jumps, allowing even lower take-off angles without slipping.

Variation of the normal force had only a small effect on the frictional behaviour of the platellae. At lower normal forces of 3 and 1 mN, platellae showed a similar direction and velocity dependence. However, the friction coefficient decreased with increasing normal forces (figure 6*c*; electronic supplementary material, tables S1–S3). For a pushing velocity of 5 mm s^−1^ and normal forces increasing from 1 to 5 mN, the friction coefficient for *Aphrodes* decreased from 2.86 to 1.00.

Friction force per contact area (shear stress) increased approximately linearly with velocity for both sliding directions (linear mixed effects model for repeated measures ANOVA: *F*_1,58_ = 281.5, *p* < 0.001; 5 mN normal force; electronic supplementary material, table S1). Shear stresses in the pulling direction appeared to be larger than in the pushing direction (*F*_1,58_ = 7.8, *p* = 0.007). However, it is likely that this result is an artefact, because as a result of the bending of the platellae, more hard tarsal spines could come into contact during pulling, thereby increasing the friction forces and leading to an overestimate of the platella shear stress.

Adhesion forces of the platellae were very small; when hind leg tarsi were pulled off perpendicularly with a speed of 0.5 mm s^−1^ after the tarsus had been in contact for 2 s, forces were below the noise level of the measurement set-up (less than 0.2 mN).

**Figure 7.**
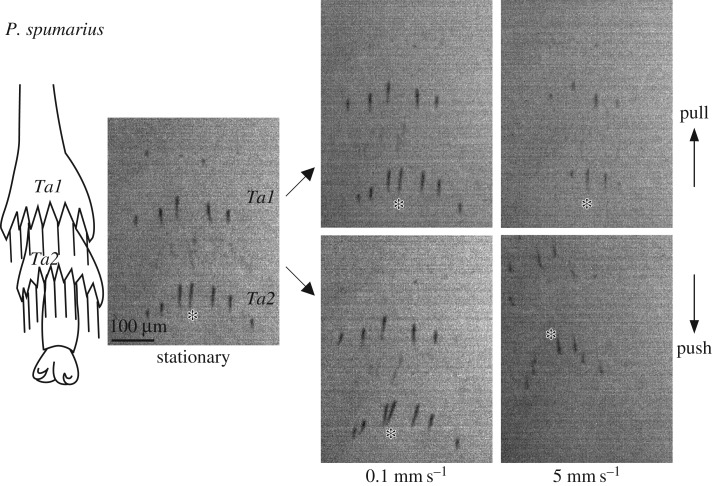
Contact area of acutellae of *Philaenus spumarius* during shear force measurements at different sliding velocities. Hind leg tarsi were brought into contact with a normal force of 5 mN (‘stationary’) and then sheared in the pushing and pulling direction for 2 s, while keeping the normal force constant. Images show contact areas 0.2 s before the end of the sliding movement. The tip of the acutella in the middle of tarsomere 2 is marked with an asterisk in all images; note that acutellae have rotated by 180° when pushed at 5 mm s^−1^. *Ta1*: tarsomere 1, *Ta2*: tarsomere 2. The scale bar applies to all images.

Although platellae are absent in *Philaenus* froghoppers, we observed that some of the thin, long acutellae protruding from behind the tarsal spines also made close contact with the substrate (figures 4*a* and 7). In stationary contact their total contact area was comparable to that of the platellae in *Aphrodes*. When pushed, however, the thin hairs bent sideways or detached, and the contact area did not increase (figures 6*e* and 7; electronic supplementary material, videos S9 and S10, and table S1; note that individual hairs rotated by almost 180° when pushed at large sliding velocities). When pulled, the hairs aligned with the direction of the movement, but the contact area even decreased at higher sliding velocities. Although friction forces of *Philaenus* tarsi weakly increased with velocity (pushing: Page's *L* test: *L*_5,8_ = 951, *p* < 0.001; pulling: *L*_5,8_ = 959, *p* < 0.001, 5 mN normal force, figure 6*d*), they were generally low, and for higher velocities in the pushing direction they were much lower than those of *Aphrodes* (5 mm s^−1^: Welch's *t*-test: *t*_4.12_ = 7.15, *p* = 0.002; 5 mN normal force; electronic supplementary material, table S1). At 5 mm s^−1^ and 5 mN normal force, the mean peak shear force in the pushing direction was 1.3 mN (*N* = 5), resulting in a friction coefficient of **μ** = 0.26, which would only allow steep upward jumps (take-off angles less than75.6°; figure 6*f*). Similar to the results for leafhoppers, the friction coefficient of *Philaenus* tarsi decreased from 0.71 to 0.26 when normal forces increased from 1 to 5 mN at a pushing velocity of 5 mm s^−1^ (figure 6*f*; electronic supplementary material, tables S1–S3).

## 4. Discussion

Our results show that *Philaenus* froghoppers and *Aphrodes* leafhoppers differ in their ability to jump from smooth surfaces. Froghoppers performed more powerful jumps than leafhoppers on the rough sandpaper surface, where the tibial and tarsal spines on their hind legs could interlock with asperities. However, they slipped on glass, resulting in jumps that were slower (four-times lower take-off velocity), had less kinetic energy (nine-times less than on sandpaper), were directed steeper upward, and had a rapid forward spin. By contrast, leafhoppers never slipped and jumped with similar take-off velocities and angles from both glass and sandpaper.

This difference in jumping ability from smooth surfaces can be explained not only by the different pushing forces produced by both insects, but also by the presence of specialized structures called platellae on the hind tarsi of leafhoppers, which are absent in froghoppers. Apart from the correlation of jumping performance with tarsal morphology, four other lines of evidence support this conclusion: first, our high-speed contact area recordings revealed that the platellae came into contact with the surface during the acceleration phase of a jump, but almost never when standing or walking. Second, platellae produced much larger contact areas than the acutellae of froghoppers, and they remained in contact during the acceleration phase (unlike the acutellae). Third, the platellae slid backward during the acceleration phase of the jump, speaking against firm interlocking. The reduction in sliding speed of the platellae without a change in contact area during the acceleration phase could reflect a variation of the insect's acceleration, but it is more likely based on the depletion of fluid from the adhesive contact zone of the platellae, similar to observations for lubricated rubber [44] or smooth and hairy adhesive pads of insects [36,45,46]. Fourth, our force measurements of leafhopper and froghopper tarsi on glass confirmed that only leafhopper platellae produced high enough friction forces to explain the observed forward jumps. The mean friction forces for platellae at 5 mN normal force would allow jumps with a take-off angle of 44.9°, close to the observed mean take-off angle of 47.7°. At 3 mN normal force, closer to the expected normal force of 3.3 mN per leg [5], the measured friction forces would allow jumps with even lower take-off angles of 35.8° (figure 6*c*). By contrast, friction forces for the tarsal hairs of *Philaenus* froghoppers at 5 mN normal force were very small, allowing only steep upward jumps with take-off angles more than 75.6°, close to the observed mean take-off angle from glass of 71.3°. Our results indicate that higher normal forces closer to the expected normal force of 12.4 mN per leg for *Philaenus* froghoppers [3] would decrease the friction coefficient (figure 6*f*), and therefore lead to even larger (steeper) minimum take-off angles.

Can the contact area of platellae observed in natural jumps of *Aphrodes* explain their jump performance on glass? Assuming a jump acceleration of 613 m s^−2^ (calculated from an acceleration time of 3.1 ms and a take-off velocity of 1.9 m s^−1^), a body mass of 18.4 mg and a take-off angle of 47.7°, the shear force can be estimated as 7.6 mN, or 3.8 mN per hind leg. With the observed mean contact area of 2484 *μ*m^2^, a shear stress of 1530 kPa would be required. This shear stress is slightly higher than the mean shear stress measured experimentally for platellae at 5 mm s^−1^ velocity and 5 mN normal force (1111 kPa). However, as the platellae slid with peak velocities of 15 to 154 mm s^−1^ during natural jumps, and as shear stresses increase with sliding velocity, it is likely that these high shear stresses were produced by the platellae. This extrapolation to higher velocities (which were experimentally unavailable) is justified, as the shear stress of rubbery pads steadily increases with velocity up to the onset of stick-slip, which we never observed for sliding platellae [47].

Platellae have been described as ‘transparent, fleshy, relatively short setae with thick and even slightly swollen, blunt, rounded tips’ [40], and were found to occur in leafhoppers (Cicadellidae) and some families of planthoppers [40–42]. The biological function of these structures has so far been unclear. Howe [41] proposed that the spines prevent the platellae from touching flat surfaces, and that they could only contribute to attachment under special conditions such as when walking on plant hairs. She also found no correlation between the number and distribution of platellae and the food plants of particular species of leafhoppers.

Our results show that platellae combine two mechanisms to control friction rapidly and reliably for jumping. The surface contact of platellae is both direction and velocity dependent: contact areas and forces were large only when the hind leg was pushed rapidly, and remained small at low pushing speeds or when pulled. This mechanism ensures good grip during the acceleration phase when the legs are pushed and easy detachment when the legs are pulled at take-off. The engagement of platellae only during the contact phase of a jump may also lessen damage and wear, which would reduce attachment performance [48].

A similar direction dependence has been reported for many insect adhesive pads [21–25,27,49]. However, the direction dependence for most attachment pads is in the opposite direction, i.e. adhesion is maximized during pulls and minimized during pushes to allow detachment. Velocity-dependent control of attachment may also be common among insect adhesive organs, but is less well documented. The shear stress of adhesive pads typically increases with sliding velocity [36,50,51]. In stick insects, higher pulling forces increase the pad's contact area (via a shear-induced change of the angle of internal cuticle fibres, [52]), and it is likely that the velocity-dependent shear force level is also responsible for the contact area change of the platellae.

The difference in function between the platellae and the adhesive arolium in leafhoppers provides a clear example of the widespread division of labour between proximal and distal attachment organs on the same foot [26,27,29,53]. The functional requirements for tarsal pads specialized for jumping are similar to those for tarsal ‘friction pads’ of non-jumping insects [30]: (i) pads should be able to deform sufficiently under load to increase contact area; (ii) when unloaded, adhesion should be minimal to allow rapid and easy detachment; (iii) pads should not buckle even when large pushing forces are acting. However, jumping pads may have to cope with even faster detachments and higher buckling forces.

The combined direction and velocity dependence of platellae may be an adaptation for the extremely rapid control of attachment required for jumping. Platellae detached in 0.2 to 0.6 ms, much faster than vertically climbing geckos (15 ms, [54]) or ants walking upside-down (more than 80 ms, [24]). Similar to many adhesive pads [23,25,52,55], the surface contact of platellae is controlled mechanically, allowing very rapid attachment and detachment. As in some tarsal friction pads [30], the adhesion forces of platellae are negligible. Thus, detachment at take-off requires only minimal force and does not slow down the jump.

The mechanical control of surface contact may be based on the morphology and orientation of the platellae: when the foot is pushed (with the spines in surface contact), a torque will develop around the base of each platella, pressing more of its soft convex side into contact and deforming it. When pulled, this torque is reversed and will help to raise the platellae away from the surface, resulting in smaller contact areas.

Our results show that the tarsal hairs (acutellae) of *Philaenus* also contact the surface during the acceleration phase of jumps. The similar anatomical position of platellae and acutellae (protruding from the dorsal surface of the base of the tarsal spines) suggests that both structures are homologous [42]. In contrast with the platellae, the acutellae only produced small pushing forces because of their instability due to bending or buckling. As a result, *Philaenus* slipped when trying to jump from glass. However, we observed that some froghopper species with more acutellae can also perform successful jumps from glass (H.H.G. and W.F. 2015, unpublished results), suggesting that even acutellae can provide grip if present in sufficient numbers.

It is still unclear to what extent the inability of *Philaenus* to jump from smooth glass affects their jumping performance on plants under natural conditions. The surfaces of most plant leaves and stems are not only more curved, but also rougher and softer than glass [56,57], so that the spines on the hind legs are more likely to find sufficient grip. It is possible that *Philaenus* avoid plants with very smooth surfaces, while *Aphrodes* can cope with a wider range, but more detailed ecological data are needed to test this hypothesis.

The hind legs of *Philaenus* are shorter than those of *Aphrodes*. As longer hind legs increase the time to accelerate and reduce the forces acting on the feet, Burrows & Sutton [6] hypothesized that long legs are beneficial on compliant substrates such as leaves. A related hypothesis is that the smaller forces for longer limbs are more favourable for the use of soft attachment structures such as platellae. A comparison of the shear forces per hind leg between both insects (*Aphrodes* 4.4 mN versus *Philaenus* 11.6 mN; [3,5]) shows that *Philaenus* produces 2.6 times more shear force while accelerating. Assuming that the shear stress of tarsal pads is approximately constant (i.e. ignoring the effect of sliding velocity), *Philaenus* would require an approximately 2.6 times larger contact area of its tarsal pads than *Aphrodes* to avoid slipping. While such larger pad areas might be anatomically possible, this difference suggests that jumping pads such as platellae are easier to realize in combination with longer legs and lower accelerations. Smaller forces for longer limbs may also expose the pads to less damage and wear, allowing the use of softer adhesive structures for attachment. However, even short-legged leafhoppers jumping with high accelerations such as *Ulopa reticulata* [6] possess platellae [58].

The adaptations for jumping from smooth surfaces reported here provide an extreme example for rapid control of surface attachment. The study of such attachment systems may help to uncover general mechanisms for switchable adhesion and inspire biomimetic synthetic devices.

## Supplementary Material

Supplementary material captions

## Supplementary Material

Supplementary Figure S1

## Supplementary Material

Supplementary Tables 1 - 3
