# Supplementary material for: Jumping without slipping: leafhoppers (Hemiptera: Cicadellidae) possess special tarsal structures for jumping from smooth surfaces
Source: J R Soc Interface. 2017 May 3;14(130):20170022. doi: 10.1098/rsif.2017.0022 (PMC5454290; doi:10.1098/rsif.2017.0022)
Supplement: Supplementary material captions [file rsif20170022supp1.pdf]

Title: Jumping without slipping: leafhoppers (Hemiptera: Cicadellidae) possess special tarsal structures for jumping from smooth surfaces

Authors: Christofer J. Clemente, Hanns Hagen Goetzke, James M. R. Bullock, Gregory P. Sutton, Malcolm Burrows, Walter Federle

Journal of the Royal Society Interface

Electronic supplementary material captions

Supplementary Figure S1: Title: Contact areas and sliding velocities in jumps by *Aphrodes* leafhoppers
Caption: Contact areas of hind leg platellae (A) and foot sliding velocities (B) in 12 jumps by 5 *Aphrodes* leafhoppers. Data from the same jumps are shown by identical colours in (A) and (B). Sliding velocity was measured by the displacement of the proximal margin of one platella contact zone. Sliding velocities were not measured when images were blurry, suggesting incomplete surface contact.

Supplementary Table S1: Title: Results of single-leg force measurements
Caption: Results of single-leg force measurements at different sliding velocities for *Aphrodes bicinctus/makarovi* leafhoppers and *Philaenus spumarius* froghoppers. The friction forces represent the peak forces during pushing or pulling slides with a normal force of 5 mN. Contact areas were measured at the point of peak force. All values are means ± s.e.m. of several insects. The sample size is given in brackets after each value.

Supplementary Table S2: Title: Results of single-leg force measurements
Caption: Results of single-leg force measurements for slides with a normal force of 3 mN, for three different velocities. All other conditions as for Supplementary Table S1.

Supplementary Table S3: Title: Results of single-leg force measurements
Caption: Results of single-leg force measurements for slides with a normal force of 1 mN, for three different velocities. All other conditions as for Supplementary Table S1.

Supplementary Video S1: Title: Side view of *Philaenus spumarius* jumping from glass
Caption: Side view of *Philaenus spumarius* jumping from glass (recorded at 4700 fps, played at 25 fps, width of field of view: 17.7 mm).

Supplementary Video S2: Title: Side view of *Philaenus spumarius* jumping from sandpaper
Caption: Side view of *Philaenus spumarius* jumping from sandpaper (recorded at 1000 fps, played at 5 fps, width of field of view: 17.3 mm).

Supplementary Video S3: Title: Side view of *Aphrodes* leafhopper jumping from glass
Caption: Side view of *Aphrodes* leafhopper jumping from glass

(recorded at 4700 fps, played at 10 fps, width of field of view: 5.5 mm).

Supplementary Video S4: Title: Side view of *Aphrodes* leafhopper jumping from sandpaper
Caption: Side view of *Aphrodes* leafhopper jumping from sandpaper (recorded at 1000 fps, played at 10 fps, width of field of view: 17.6 mm).

Supplementary Video S5: Title: Hind foot contact area recording of *Aphrodes* leafhopper jumping from glass
Caption: Hind foot contact area recording of *Aphrodes* leafhopper jumping from glass (recorded at 5000 fps, played at 3 fps, width of video: 935 µm).

Supplementary Video S6: Title: Hind foot contact area recording of *Philaenus spumarius* jumping from glass
Caption: Hind foot contact area recording of *Philaenus spumarius* jumping from glass (recorded at 4700 fps, played at 3 fps, width of video: 431 µm).

Supplementary Video S7: Title: Contact area recording during force measurement of *Aphrodes* leafhopper
Caption: Contact area recording during force measurement of *Aphrodes* leafhopper. The tarsus is brought into contact with a feedback-controlled normal force of 5 mN. The tarsus is first sheared in the pushing direction with a velocity of 0.1 mm/s for 2 s. The tarsus is then detached and brought back into contact with the same normal force, followed by shearing in the pulling direction with the same velocity of 0.1 mm/s and subsequent detachment (recorded at 10 fps, played at 10 fps, width of video: 513 µm).

Supplementary Video S8: Title: Contact area recording during force measurement of *Aphrodes* leafhopper
Caption: Contact area recording during force measurement of *Aphrodes* leafhopper. Conditions as in supplementary video S7, but with a velocity of 5.0 mm/s (recorded at 10 fps, played at 10 fps, width of video: 513 µm).

Supplementary Video S9: Title: Contact area recording during force measurement of *Philaenus spumarius*
Caption: Contact area recording during force measurement of *Philaenus spumarius*. Conditions as in supplementary video SS7 (recorded at 10 fps, played at 10 fps, width of video: 811 µm).

Supplementary Video S10: Title: Contact area recording during force measurement of *Philaenus spumarius*
Caption: Contact area recording during a force measurement of *Philaenus spumarius*. Conditions as in supplementary video 7, but with a velocity of 5.0 mm/s (recorded at 10 fps, played at 10 fps, width of video: 811 µm).
