# Supplementary figures and images for: Jumping without slipping: leafhoppers (Hemiptera: Cicadellidae) possess special tarsal structures for jumping from smooth surfaces
Source: J R Soc Interface. 2017 May 3;14(130):20170022. doi: 10.1098/rsif.2017.0022 (PMC5454290; doi:10.1098/rsif.2017.0022)

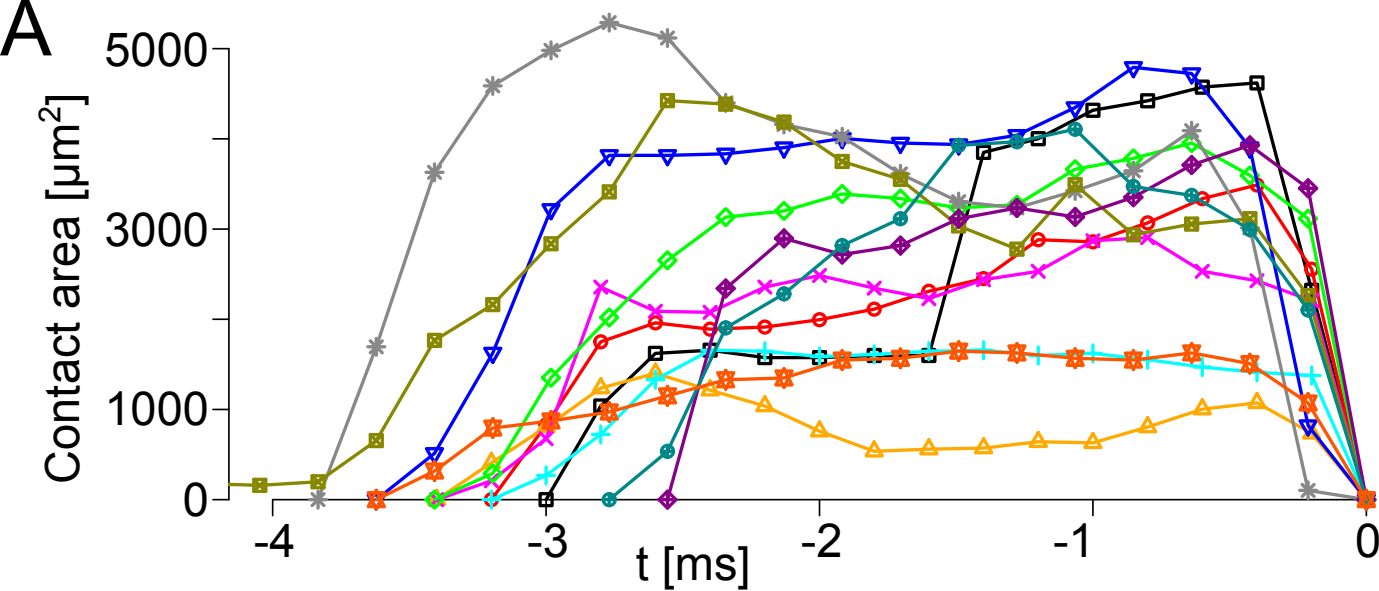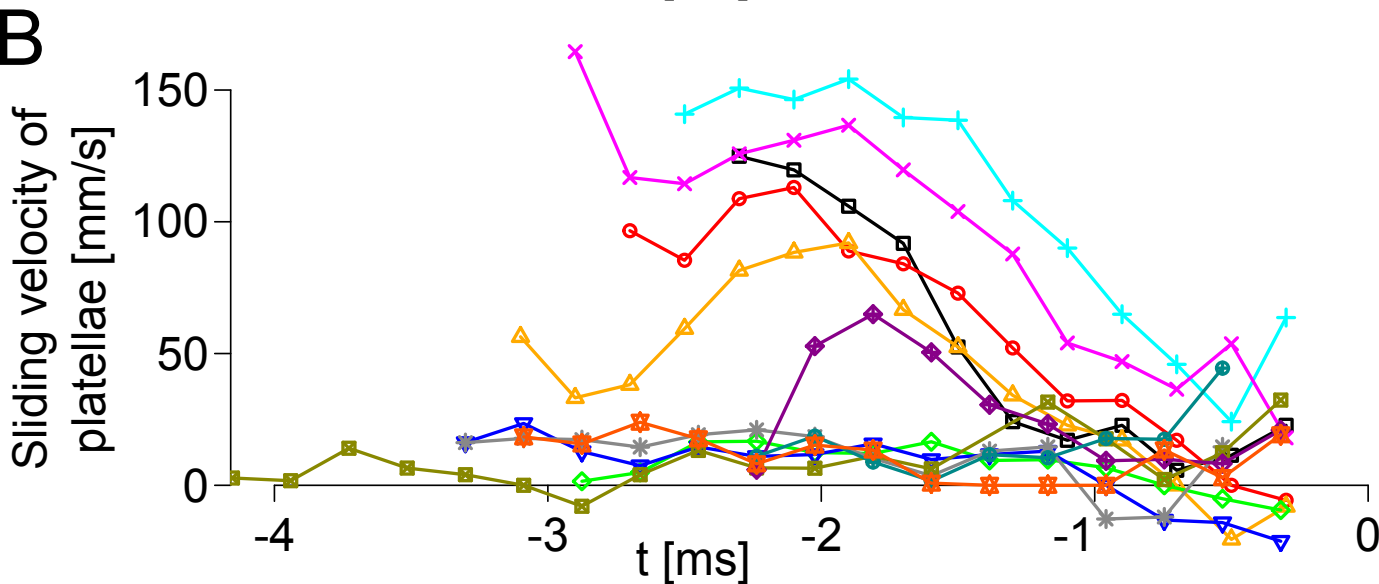

Supplement: Supplementary Figure S1 [file rsif20170022supp2.pdf]
