# Supplementary material for: Jumping without slipping: leafhoppers (Hemiptera: Cicadellidae) possess special tarsal structures for jumping from smooth surfaces
Source: J R Soc Interface. 2017 May 3;14(130):20170022. doi: 10.1098/rsif.2017.0022 (PMC5454290; doi:10.1098/rsif.2017.0022)
Supplement: Supplementary Tables 1 - 3 [file rsif20170022supp3.pdf]

**Journal of the Royal Society Interface**

# Title: Jumping without slipping: leafhoppers (Hemiptera: Cicadellidae) possess special tarsal structures for jumping from smooth surfaces

**Authors: Christofer J. Clemente, Hanns Hagen Goetzkea, James M. R. Bullock, Gregory P. Sutton, Malcolm Burrows, Walter Federle**

Table 1: **Results of single leg force measurements at different sliding velocities for *Aphrodes bicinctus/makarovi* leafhoppers and *Philaenus spumarius* froghoppers (slides with a normal force of 5 mN; values are mean ± s.e.m. (N))**

| | | **Sliding velocity [mm/s]** | 0.1 | 0.2 | 0.5 | 0.7 | 1.0 | 2.0 | 3.0 | 5.0 |
|---|---|---|---|---|---|---|---|---|---|---|
| *Aphrodes bicinctus/makarovi* | **Pushing direction** | Friction [mN] | $0.64 \pm 0.05(4)$ | $0.71 \pm 0.07(4)$ | $0.90 \pm 0.02(4)$ | $1.04 \pm 0.03(4)$ | $1.39 \pm 0.13(4)$ | $2.47 \pm 0.17(4)$ | $3.35 \pm 0.27(4)$ | $5.01 \pm 0.48(4)$ |
| | | Contact area [µm$^2$] | $1591.3 \pm 279.2(4)$ | $1669.9 \pm 234.6(4)$ | $2032.8 \pm 284.6(4)$ | $2406.5 \pm 311.7(4)$ | $2868.4 \pm 481.7(4)$ | $3799.3 \pm 533.6(4)$ | $4448.6 \pm 678.9(4)$ | $4595.8 \pm 595.5(4)$ |
| | | Shear stress [kPa] | $456.9 \pm 120.6(4)$ | $474.5 \pm 115.6(4)$ | $475.6 \pm 85.4(4)$ | $447.5 \pm 45.0(4)$ | $533.0 \pm 87.1(4)$ | $672.6 \pm 61.8(4)$ | $785.7 \pm 76.5(4)$ | $1110.5 \pm 72.1(4)$ |
| | | Friction coefficient $\mu$ | 0.13 | 0.14 | 0.18 | 0.21 | 0.28 | 0.49 | 0.67 | 1.00 |
| | | Minimal take-off angle $\alpha$ [deg] | 82.7 | 81.9 | 79.8 | 78.3 | 74.5 | 63.7 | 56.2 | 44.9 |
| | **Pulling direction** | Friction [mN] | $0.62 \pm 0.06(4)$ | $0.59 \pm 0.09(4)$ | $0.70 \pm 0.09(4)$ | $0.70 \pm 0.12(4)$ | $0.83 \pm 0.06(4)$ | $1.06 \pm 0.07(4)$ | $1.22 \pm 0.15(4)$ | $1.44 \pm 0.22(4)$ |
| | | Contact area [µm$^2$] | $1187.8 \pm 183.7(4)$ | $1221.4 \pm 179.6(4)$ | $1194.5 \pm 180.2(4)$ | $1187.5 \pm 251.1(4)$ | $1175.1 \pm 153.7(4)$ | $1051.8 \pm 208.2(4)$ | $1150.1 \pm 223.5(4)$ | $905.2 \pm 168.3(4)$ |
| | | Shear stress [kPa] | $606.4 \pm 187.8(4)$ | $544.3 \pm 164.8(4)$ | $661.9 \pm 186.7(4)$ | $705.1 \pm 209.2(4)$ | $837.5 \pm 161.0(4)$ | $1105.3 \pm 201.3(4)$ | $1152.7 \pm 236.3(4)$ | $1725.0 \pm 276.6(4)$ |
| *Philaenus spumarius* | **Pushing direction** | Friction [mN] | $0.67 \pm 0.09(5)$ | $0.67 \pm 0.09(5)$ | $0.74 \pm 0.07(5)$ | $0.80 \pm 0.09(5)$ | $0.87 \pm 0.07(5)$ | $1.01 \pm 0.09(5)$ | $1.15 \pm 0.13(5)$ | $1.28 \pm 0.21(5)$ |
| | | Contact area [µm$^2$] | $2720.4 \pm 568.0(5)$ | $2166.1 \pm 473.9(5)$ | $2428.5 \pm 530.8(5)$ | $2377.6 \pm 649.5(5)$ | $2474.5 \pm 438.3(5)$ | $2394.0 \pm 402.7(4)$ | $1842.1 \pm 419.1(5)$ | $2218.2 \pm 566.9(4)$ |
| | | Shear stress [kPa] | $322.0 \pm 99.7(5)$ | $365.9 \pm 85.7(5)$ | $367.4 \pm 90.5(5)$ | $495.0 \pm 198.1(5)$ | $455.2 \pm 103.7(5)$ | $446.4 \pm 63.4(4)$ | $749.8 \pm 175.9(5)$ | $619.0 \pm 158.9(4)$ |
| | | Friction coefficient $\mu$ | 0.13 | 0.13 | 0.15 | 0.16 | 0.17 | 0.20 | 0.23 | 0.26 |
| | | Minimal take-off angle $\alpha$ [deg] | 82.4 | 82.4 | 81.6 | 80.9 | 80.1 | 78.6 | 77.0 | 75.6 |
| | **Pulling direction** | Friction [mN] | $0.64 \pm 0.06(5)$ | $0.65 \pm 0.07(5)$ | $0.72 \pm 0.10(5)$ | $0.77 \pm 0.09(5)$ | $0.79 \pm 0.09(5)$ | $0.91 \pm 0.12(5)$ | $0.97 \pm 0.15(5)$ | $1.14 \pm 0.19(5)$ |
| | | Contact area [µm$^2$] | $2200.3 \pm 464.4(5)$ | $2094.1 \pm 474.0(5)$ | $1854.3 \pm 420.9(5)$ | $1715.8 \pm 362.0(5)$ | $1953.0 \pm 327.7(5)$ | $1184.3 \pm 169.6(5)$ | $1138.2 \pm 251.5(4)$ | $954.7 \pm 170.3(5)$ |
| | | Shear stress [kPa] | $357.9 \pm 89.6(5)$ | $354.1 \pm 56.2(5)$ | $442.6 \pm 61.8(5)$ | $511.3 \pm 84.7(5)$ | $512.0 \pm 63.7(5)$ | $784.5 \pm 53.9(5)$ | $966.2 \pm 85.9(4)$ | $1241.8 \pm 168.7(5)$ |

Table 2: **Results of single leg force measurements at different sliding velocities for *Aphrodes bicinctus/makarovi* leafhoppers and *Philaenus spumarius* froghoppers (slides with a normal force of 3 mN; values are mean ± s.e.m. (N))**

| | | **Sliding velocity [mm/s]** | 1.0 | 3.0 | 5.0 |
|---|---|---|---|---|---|
| *Aphrodes bicinctus/makarovi* | **Pushing direction** | Friction [mN] | $1.03 \pm 0.17(4)$ | $2.83 \pm 0.44(4)$ | $4.15 \pm 0.44(4)$ |
| | | Contact area [µm$^2$] | $2492.8 \pm 518.0(4)$ | $3750.0 \pm 611.4(4)$ | $4040.3 \pm 495.0(4)$ |
| | | Shear stress [kPa] | $441.9 \pm 57.5(4)$ | $764.7 \pm 33.3(4)$ | $1037.6 \pm 35.5(4)$ |
| | | Friction coefficient $\mu$ | 0.34 | 0.94 | 1.39 |
| | | Minimal take-off angle $\alpha$ [deg] | 71.2 | 47.6 | 36.4 |
| | **Pulling direction** | Friction [mN] | $0.61 \pm 0.07(4)$ | $0.84 \pm 0.13(4)$ | $1.12 \pm 0.23(4)$ |
| | | Contact area [µm$^2$] | $764.4 \pm 51.1(4)$ | $1110.1 \pm 399.0(4)$ | $788.4 \pm 116.1(4)$ |
| | | Shear stress [kPa] | $799.0 \pm 58.4(4)$ | $923.5 \pm 190.3(4)$ | $1385.7 \pm 153.3(4)$ |
| *Philaenus spumarius* | **Pushing direction** | Friction [mN] | $0.70 \pm 0.10(5)$ | $0.91 \pm 0.15(5)$ | $1.03 \pm 0.20(5)$ |
| | | Contact area [µm$^2$] | $2604.4 \pm 557(4)$ | $2276.6 \pm 681.5(4)$ | $1972.6 \pm 691.9(4)$ |
| | | Shear stress [kPa] | $352.6 \pm 76.2(4)$ | $552.8 \pm 116.9(4)$ | $1303.5 \pm 779.6(4)$ |
| | | Friction coefficient $\mu$ | 0.23 | 0.30 | 0.34 |
| | | Minimal take-off angle $\alpha$ [deg] | 76.9 | 73.3 | 71.3 |
| | **Pulling direction** | Friction [mN] | $0.62 \pm 0.08(5)$ | $0.77 \pm 0.13(5)$ | $0.86 \pm 0.13(5)$ |
| | | Contact area [µm$^2$] | $1869.3 \pm 511.2(4)$ | $1082.0 \pm 358.4(4)$ | $1002.5 \pm 247.2(4)$ |
| | | Shear stress [kPa] | $428.9 \pm 96.0(4)$ | $884.5 \pm 116.2(4)$ | $1130.1 \pm 240.0(4)$ |

Table 3: **Results of single leg force measurements at different sliding velocities for *Aphrodes bicinctus/makarovi* leafhoppers and *Philaenus spumarius* froghoppers (slides with a normal force of 1 mN; values are mean ± s.e.m. (N))**

| | | **Sliding velocity [mm/s]** | 1.0 | 3.0 | 5.0 |
|---|---|---|---|---|---|
| *Aphrodes bicinctus/makarovi* | **Pushing direction** | Friction [mN] | $0.81 \pm 0.13(3)$ | $2.08 \pm 0.24(3)$ | $2.87 \pm 0.46(3)$ |
| | | Contact area [µm$^2$] | $2419.3 \pm 463.9(3)$ | $3082.9 \pm 278.5(3)$ | $2992.0 \pm 456.7(3)$ |
| | | Shear stress [kPa] | $340.2 \pm 23.2(3)$ | $672.8 \pm 18.3(3)$ | $957.3 \pm 44.6(3)$ |
| | | Friction coefficient $\mu$ | 0.81 | 2.08 | 2.86 |
| | | Minimal take-off angle $\alpha$ [deg] | 51.6 | 26.1 | 20.0 |
| | **Pulling direction** | Friction [mN] | $0.36 \pm 0.06(3)$ | $0.63 \pm 0.12(3)$ | $0.75 \pm 0.13(3)$ |
| | | Contact area [µm$^2$] | $903.9 \pm 97.1(3)$ | $866.7 \pm 184.0(3)$ | $762.2 \pm 271.7(3)$ |
| | | Shear stress [kPa] | $837.5 \pm 161.0(3)$ | $1105.3 \pm 201.3(3)$ | $1152.7 \pm 236.3(3)$ |
| *Philaenus spumarius* | **Pushing direction** | Friction [mN] | $0.40 \pm 0.08(5)$ | $0.62 \pm 0.15(5)$ | $0.71 \pm 0.16(5)$ |
| | | Contact area [µm$^2$] | $2076.3 \pm 709.5(4)$ | $1863.2 \pm 540.1(4)$ | $1776.1 \pm 681.5(4)$ |
| | | Shear stress [kPa] | $311.3 \pm 111.6(4)$ | $450.1 \pm 73.4(4)$ | $713.3 \pm 229.8(4)$ |
| | | Friction coefficient $\mu$ | 0.40 | 0.62 | 0.71 |
| | | Minimal take-off angle $\alpha$ [deg] | 68.8 | 59.6 | 56.3 |
| | **Pulling direction** | Friction [mN] | $0.36 \pm 0.07(5)$ | $0.46 \pm 0.08(5)$ | $0.50 \pm 0.07(5)$ |
| | | Contact area [µm$^2$] | $1699.9 \pm 567.5(4)$ | $893.1 \pm 189.7(4)$ | $1265.8 \pm 531.8(4)$ |
| | | Shear stress [kPa] | $320.0 \pm 88.0(4)$ | $625.8 \pm 77.2(4)$ | $787.5 \pm 321.4(4)$ |
